# Recent Advances in Biomolecule–Nanomaterial Heterolayer-Based Charge Storage Devices for Bioelectronic Applications

**DOI:** 10.3390/ma13163520

**Published:** 2020-08-10

**Authors:** Taek Lee, Soomin Kim, Jinmyeong Kim, Sang-Chan Park, Jinho Yoon, Chulhwan Park, Hiesang Sohn, Jae-Hyuk Ahn, Junhong Min

**Affiliations:** 1 Department of Chemical Engineering, Kwangwoon University, Seoul 01897, Korea; k-soomin@hotmail.com (S.K.); wls629@icloud.com (J.K.); chpark@kw.ac.kr (C.P.); hsohn@kw.ac.kr (H.S.); 2 Department of Electronic Engineering, Kwangwoon University, Wolgye-dong, Nowon-gu, Seoul 01899, Korea; qkrtkdcks701@kw.ac.kr; 3 Department of Chemistry and Chemical Biology, Rutgers, The State University of New Jersey, Piscataway, NJ 08854, USA; iverson0607@naver.com; 4 School of Integrative Engineering, Chung-Ang University, Seoul 06974, Korea

**Keywords:** biomemristor, field-effect transistor, biomolecule, nanomaterial, charge storage device, heterolayer

## Abstract

With the acceleration of the Fourth Industrial Revolution, the development of information and communications technology requires innovative information storage devices and processing devices with low power and ultrahigh stability. Accordingly, bioelectronic devices have gained considerable attention as a promising alternative to silicon-based devices because of their various applications, including human-body-attached devices, biomaterial-based computation systems, and biomaterial–nanomaterial hybrid-based charge storage devices. Nanomaterial-based charge storage devices have witnessed considerable development owing to their similarity to conventional charge storage devices and their ease of applicability. The introduction of a biomaterial-to-nanomaterial-based system using a combination of biomolecules and nanostructures provides outstanding electrochemical, electrical, and optical properties that can be applied to the fabrication of charge storage devices. Here, we describe the recent advances in charge storage devices containing a biomolecule and nanoparticle heterolayer including (1) electrical resistive charge storage devices, (2) electrochemical biomemory devices, (3) field-effect transistors, and (4) biomemristors. Progress in biomolecule–nanomaterial heterolayer-based charge storage devices will lead to unprecedented opportunities for the integration of information and communications technology, biotechnology, and nanotechnology for the Fourth Industrial Revolution.

## 1. Introduction

In the World Economic Forum at Davos, Prof. Klaus Schwab declared that the Fourth Industrial Revolution began in 2015 [1]. Accordingly, technical integration has been accelerated. For example, information technology integrated with communication technology that gave information and communications technology (ICT). In particular, emerging biotechnology (BT), nanotechnology (NT), and ICT have created new technological industries such as remote medicine [2], delicate drug-delivery system [3], artificial-intelligence-based diagnosis [4], and internet of things (IoT)-based smart healthcare systems [5]. These fields contribute to life extension of human, intractable disease treatment, advancement of medical service for people. Among them, the integration of biotechnology and electrical engineering has produced bioelectronics [6,7]. Bioelectronics has led to the development of new devices that can be applied to in vitro diagnostics [8], biosensors [9], biochips [10], and biocomputing devices [11].

Particularly, bioelectronic devices such as memory, processors, transducers, charge storage devices, field-effect transistors, logic gates, and memristors have been developed [12,13,14]. Bioelectronic devices are usually composed of biomaterials and nanomaterials that resemble silicon-based electronic computation elements [14]. These bioelectronic devices were developed based on the following objectives: (1) These bioelectronic devices show electrical properties better than or similar to those of silicon-based electronic devices [15]. (2) Bioelectronic devices composed of biomaterials and nanomaterials are prepared with alternatives of the elements of silicon-based electronic devices [16]. (3) These bioelectronic devices show new functionalities such as cell-based biocomputation, molecular computation, brain computation, and electrochemical computation [17,18,19]. (4) Bioelectronic devices have been developed for biomedical engineering applications, such as implantable devices, wearable devices, and bioelectronic medicine [20,21,22]. These objectives are difficult to achieve with current silicon-based electronic devices. Particularly, charge storage devices are important bioelectronic devices because they can be extended to various information storage devices such as random-access memory [23], multi-level memory [24], flash memory [25], resistive memory [26], and memristors [27]. Recently, bioelectronic devices have been applied to new biomedical engineering areas that could not be served with existing biomedical engineering applications. For example, implantable bioelectronic devices offer new opportunities to patients with incurable nerve system disorders [20,21,28,29]. Compared the organic molecule-based charge storage device, the biomolecule-based charge storage device can be easily attached to living organism skin. Especially, the bioelectronic medicine device to treat the degenerative neuronal diseases that can stimulate the nerve system through electron pulses from fabricated device [20,28].

**Figure 1:**
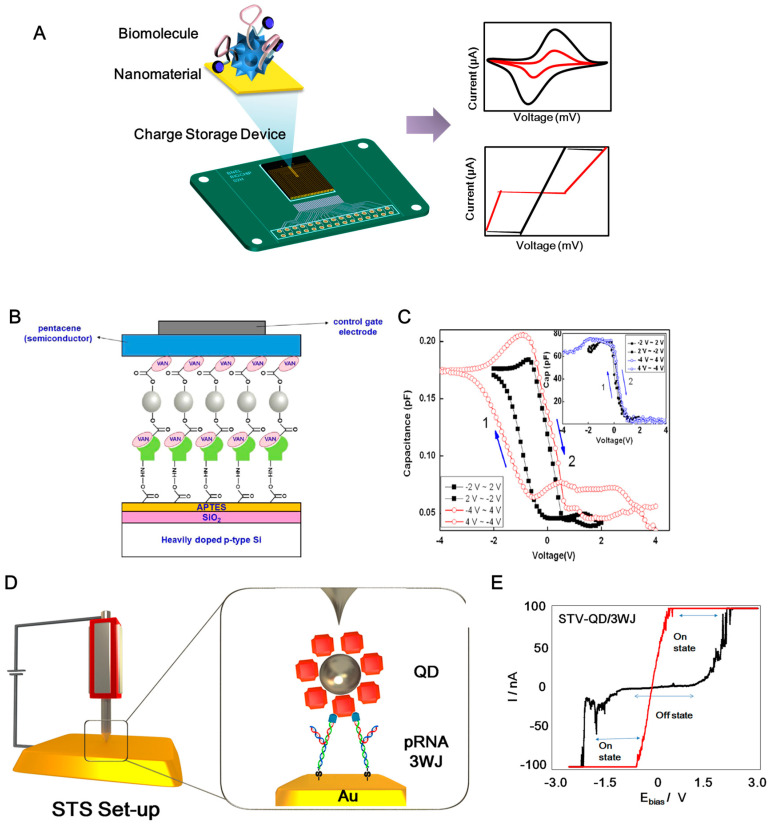
(**A**) General structure of charge storage device composed of biomolecule and nanomaterial heterolayer. (**B**) Schematic diagram of process to fabricate metal–pentacene–insulator–silicon (MPIS) device structure. (**C**) *C*–*V* characteristics of VAN conjugated ZnO NPs embedded MPIS device measured at 100 kHz and an inlet graph showing the *C*–*V* curves of a control MPIS device without ZnO NPs embedding. Reproduced with permission from [44], published by Elsevier, 2014. (**D**) Schematic diagram for constitution STV/QD-RNA 3WJ conjugates on Au substrate. (**E**) *I*–*V* characterization of the electrical bi-stability behavior. *I*–*V* characteristics of the STV/QD/RNA 3WJ chimera on Au substrate, exhibiting electrical bi-stability behavior where the current transition from the low (OFF state) to high (ON state) and from low resistance state to high resistance showing the charge storage effect. Reproduced with permission from [25], published by American Chemical Society, 2015.

Combining biomaterials and nanomaterials can yield unprecedented functionalities in bioelectronic devices for application to future biocomputation systems [12,16]. Various biomaterials—such as metalloproteins, antibodies, enzymes, DNA, RNA, ribozymes, and aptamers—have been employed to develop bioelectronic devices [15,30,31,32,33]. These biomaterials not only use direct internal properties but also embody the new functionality via recombinant technique or external modification [23,25]. Furthermore, the introduction of nanotechnology has extended the application of biomaterials toward the development of bioelectronic devices. Nanotechnology has improved the advancement of bioelectronic devices in two ways. First, nanotechnology provides nanopatterns, nanostructures, and nanoscale electrodes that can be fabricated via lithography [34,35,36]. The fabricated nanopatterns can be used as the electrode platform. Second, nanotechnology yields synthesized nanomaterials—such as carbon nanotubes, graphene, gold nanoparticles (AuNPs), silver nanoparticles, quantum dots (QDs), and topological insulators [37,38]. The synthesized nanomaterial can be combined with biomaterials to enhance their electrical properties or create new functionalities. Recently, bioelectronics has paved the way toward the development of eco-friendly electronic devices i.e., “green electronics” [39,40]. Green electronics has provided devices that can be degraded after their use. These techniques have resulted in the advancement of the new concept of charge storage devices containing biomolecules and nanomaterials heterolayer. The biomolecule/nanomaterial heterolayer was defined as the combination of biomaterial including protein, nucleic acid, and other biologically oriented material and nanomaterial including particles, structures, and technology. Figure 1A showed the schematic diagram for general structure of charge storage device composed of biomolecule/nanomaterial heterolayer. The present review discusses the recent progress in bioelectronic devices composed of biomolecules and nanomaterial heterolayers for charge storage applications.

## 2. Electrical Charge Storage Device

An electrical charge storage device composed of a biomolecule/nanomaterial heterolayer on a substrate is usually used as a vertical structure for the hysteresis of the heterolayer [33]. When an appropriate potential was applied to a biomolecule/nanomaterial heterolayer on the metal substrate, the current response showed a charge storage and discharge effect. In these cases, the biomolecules acted as an insulator and the nanomaterial acted as a conductor or semiconductor. The combination of biomolecules and nanomaterials exhibited various hysteresis effects, which can be used to construct charge storage devices [33,41,42,43].

Kim et al. fabricated an electrical charge storage device consisting of ZnO nanoparticles conjugated with vancomycin (VAN) using an electrical charging node. VAN-conjugated ZnO nanoparticles acted as the charge storage elements in a metal–pentacene–insulator–silicon (MPIS) hybrid structure [44]. Usually, VAN was used to treat the antibacterial reagent and ZnO nanoparticle usually applied to sunscreen cream and fuel cell components. However, this study developed the electrical charge storage device composed of VAN/ZnO nanoparticle heterolayer. The electrical conduction and charging between VAN and ZnO could be modulated by the bottom (SiO_2_) and top (pentacene) electrodes. In addition, for anchoring VAN onto the SiO_2_ substrate, the peptide (L-Ala-D-Glu-L-Lys-D-Ala-D-Ala), which can recognize vancomycin, was introduced as the linker (Figure 1A). The electrical properties of the fabricated VAN-ZnO MPIS structure were validated using a capacitance versus voltage (*C–V*) curve that showed flat-band voltage shifts. The hysteresis of the proposed nanobio hybrid structure demonstrated the charge storage function of the fabricated MPIS structure. Figure 1B shows the *C–V* results of the MPIS capacitor device, which displayed features of a nonvolatile charge storage device at a frequency of 100 kHz. The inset graph of Figure 1C shows the *C–V* curves of a control MPIS device without the formation of VAN-conjugated ZnO NPs. Thus, the MPIS structure with VAN only showed good hysteresis. Furthermore, the retention time of charge storage was 10^5^ s. In addition, an MPIS structure containing DNA A10 aptamers and AuNPs was proposed for use in charge storage devices [45]. Azurin and CdSe-ZnS QD heterolayers were used to fabricate a resistive-type charge storage device [46]. To induce hysteresis, a CdSe-ZnS QD was immobilized on a Au surface via 1-4 dithiane. Then, azurin was anchored on a QD-modified substrate via a 1-octadecanethiol linker. This metal–semiconductor–insulator structure displayed good hysteresis. Some research groups have introduced virus and nanoparticle heterostructures for charge storage devices [47,48]. Tobacco mosaic virus conjugated with platinum nanoparticles was directly used in digital charge storage devices [47]. In addition, the recombinant cowpea mosaic virus (CPMV) conjugated with CdSe-ZnS QD on an Au substrate showed a nanoscale charge storage effect [48]. In this case, the CPMV acted as an insulator and QD acted as a semiconductor in the fabrication of metal–insulator–semiconductor (MIS) structures. The conductive atomic force microscopy (CAFM) with contact mode under the open-air condition was used to investigate the *I–V* characteristics of the single MIS structure for validation of the nanoscale charge storage effect. For this, the CAFM tip was directly fixed on the single structure and then, the voltage was applied to the contacted single MIS structure for acquiring the *I–V* characteristics. From results, the combination of viral proteins and nanomaterial showed interesting characteristics for charge storage devices.

Furthermore, DNA/nanomaterial hybrid structures have been applied to develop electrical charge storage devices [25,49,50,51]. A resistive switching device consisting of carboxyl-modified MoS_2_ and a thiol-modified DNA heterolayer was proposed [51]. The metal dichalcogenide MoS_2_ is considered a new semiconducting material because of its unique features such as direct band gap, conductivity, and biocompatibility. Several studies have reported the application of MoS_2_ for capacitors and batteries [52,53,54,55]. Here, a thiol-modified single-stranded DNA layer was anchored on the Au substrate. Then, amine-modified complementary DNA was hybridized with the thiol-modified DNA layer. Then, carboxyl-MoS_2_ was reacted with the 1-ethyl-3-(3-dimethylaminopropyl) carbodiimide hydrochloride (EDC)/N-hydroxysuccinimide (NHS) coupling reagent to fabricate the MIS structure. The electrical switching properties of MoS_2_-DNA on the Au substrate were validated via scanning tunneling spectroscopy (STS) experiments. Compared with the DNA and MoS_2_ layers, the MoS_2_-DNA heterolayer showed excellent hysteresis. The hysteresis of the MoS_2_-DNA layer was retained for 50 cycles.

Previous studies have only shown the properties of biomolecule–nanomaterial heterolayers. It is difficult to determine whether the origin of hysteresis is the sum of the biomolecule/nanomaterial heterolayer or the biomolecule/nanomaterial conjugate. To determine the hysteresis effect, Guo’s group presented a nanoscale electrical charge storage device composed of an RNA and QD chimera structure [25]. Conventionally, RNA molecules are regarded as unstable materials because of their delicate structure and the presence of several RNases, which limits the use of RNA molecules as building materials for electrical devices. To overcome this problem, the authors introduced a packaging RNA three-way junction (3WJ). The thermodynamic stability of a pRNA 3WJ from bacteriophage phi29 DNA packaging motor was reported, which can be used for various drug delivery and cancer therapy applications [56,57,58,59]. By introducing the RNA 3WJ, nanoscale charge storage composed of an RNA-QD chimera structure was developed (Figure 1D). QD showed the outstanding fluorescent effect that can be usually applied to biomedical imagining and display panel. Introducing the QD to RNA exhibited the interesting electrical bistability. The authors introduced an MIS structure to demonstrate a resistive charge storage function (Figure 1E). To construct the MIS structure at the nanoscale, a streptavidin-coated QD was conjugated with biotin, sephadex-aptamer-tagged RNA 3WJ based on site-specific conjugation. After the site-specific conjugation step, the prepared RNA-QD fragment was reassembled with the thiol-tagged RNA fragment. Then, the thiol-modified RNA-QD chimera structure was immobilized on the Au substrate. STS was used to validate the charge storage function (Figure 1C). STS can adjust the tip position to the RNA-QD on the Au substrate. When the voltage was applied from the STS tip to the RNA-QD, the current with resistive property was monitored and applied to the charge storage device. The current–voltage relationship in the OFF state showed the injection-dominated thermionic emission model. In addition, the current–voltage relationship in the ON state can be explained by the space-charge limited conduction emission mechanism. The on/off current ratio was 100 (Figure 1E). The fabricated RNA-QD structure exhibited resistive memory properties for two weeks. After two weeks, the bistability-resistive property was not observed in the RNA-QD substrate which might be the damage of RNA-QD heterolayer. This study demonstrated the origin of hysteresis through the RNA-QD chimera structure [25]. Thus, the tailored RNA and QD hybrid material can be used for charge storage applications.

## 3. FET Devices

A field-effect transistor (FET), as a logic or memory device, is an important building block for constructing electronic circuits. Compared with modern electronic devices based on silicon technology, biomolecule-based FETs have many advantages in manufacturing and disposal because they are biodegradable, biocompatible, environmentally friendly, and naturally abundant, thus reducing electronic waste [60]. The integration of biomolecules with nanoparticles provides a new class of hybrid nanomaterials with a combination of their unique properties, revealing new tailored and synergistic functionalities [61]. The electron transfer characteristics of biomolecules and nanoparticles allow the storage of charge for memory devices [62,63]. In this section, we will introduce significant works related to FETs or memory devices, in which the biomolecule–nanoparticle hybrids function as (i) channels, (ii) gate dielectrics, and (iii) functionalized charge-trapping layers.

**Figure 2:**
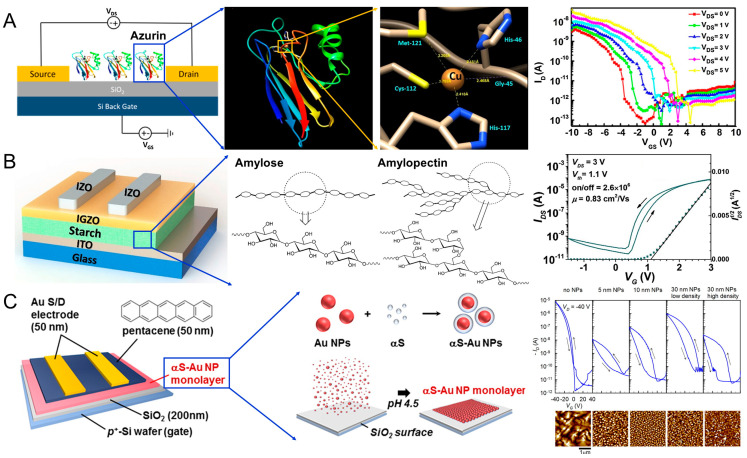
(**A**) Schematic illustration of azurin-based FET. Crystal structure (generated by UCSF Chimera, https://www.cgl.ucsf.edu/chimera) of native *Pseudomonas aeruginosa* azurin. Structural depiction of the metal site in native azurin (PDB ID: 1AZU). Cu-azurin FET—Drain current versus gate to source voltage with drain to source voltage varying between 0 to 5 V in steps. Reproduced with permission from [64], published by Elsevier, 2017. (**B**) Device structure of the thin film transistors with starch as the ion-based gate dielectric. Basic structural motifs of amylose and amylopectin. Transfer curves of IGZO transistors gated by starch dielectrics of glycerol incorporated potato starch. Reproduced with permission from [69], published by Elsevier, 2017. (**C**) Structure of the organic FET-based memory with protein-NP conjugate monolayer structure. Schematic representation of the αS–Au NP monolayer formation on SiO_2_. Specific interaction of αS (blue) with Au NPs (red) produces the homogeneous conjugates of αS−Au NPs (upper row). Buffer change from pH 6.5 to 4.5 induces adsorption onto SiO_2_, leading to the closely packed monolayer (lower row). Dual-sweep transfer curves for different αS–Au NP structures. Corresponding AFM images of the 50 nm thick pentacene channel are presented below. Adapted with permission from [72]. Copyright 2016 American Chemical Society.

Kalyani et al. utilized *Pseudomonas aeruginosa* azurin as a channel (200 nm) between the source and drain electrodes, as shown in Figure 2A [64]. The azurin has the redox property that investigated to understand the electron transfer property in the living organism and cancer therapy. However, azurin can be used to FET construction as the building material. The electron transfer capability and redox activity of the protein could be tuned by an external potential, which results in FET behavior [65,66]. The copper ions present in the redox active site make the protein resistant to thermal denaturation (*T_m_* = 86 °C) [67]. The fabricated devices showed *p*-type characteristics with tunable electrical parameters such as sub-threshold swing and on/off ratio by modifying the metal ion in the redox active site (Figure 2A). The azurin-based FET exhibited good thermal stability up to 70 °C. Moudgil et al., demonstrated an azurin–TiO_2_ hybrid nanostructure FET in which *Pseudomonas aeruginosa* azurin proteins were immobilized on TiO_2_ film of thickness 80 nm [68]. Compared with azurin and TiO_2_ devices, the azurin–TiO_2_ hybrid device showed more than three times the spectral responsivity for UV wavelengths. This is attributed to the synergetic effects of the metal oxide and azurin, such as the efficient separation of the electron–hole pair due to the excellent heterostructure and the improved channel conductivity with interlayer resistance and capacitance.

Shao et al. utilized two types of starches—i.e., water-soluble starch and potato starch—as ion-based gate dielectrics for indium gallium zinc oxide (IGZO)-based FETs, as shown in Figure 2B [69]. The device with glycerol-incorporated potato starch exhibited a high on/off ratio (2.6 × 10^6^) and field-effect mobility (0.83 cm^2^ V^−1^ s^−1^) due to high capacitance and ion conductivity (Figure 2B). Water-soluble starch with lower ion conductivity caused a large current hysteresis for potential application as a memory device. Chiu et al. demonstrated glucose-based oligo- and polysaccharides (hydrophilic biopolymers) as the charge-storage layer in FET-based memory devices [70]. A thin film (~30 nm) of the oligosaccharide maltoheptaose was inserted between a 50 nm-thick semiconducting pentacene thin film and a 100 nm-thick SiO_2_ layer. The maltoheptaose layer provided strong electron-trapping properties owing to the charged hydroxyl groups to form strong hydrogen bonds, which facilitates the stabilization of the trapped charges. Liang et al. employed DNA molecules attached with a long chain of octadecyltrimethylammonium (OTMA) as the gate dielectric for charge storage elements in FET-based memory devices [71]. In response to an applied gate voltage, movable dipoles existing in the DNA–OTMA complex tend to polarize along the electric field to store charges by increasing the accumulated carriers in the semiconducting channel.

Kim et al. reported a charge-trapping layer based on protein-mediated AuNPs with a precise control over the dimension and distribution of organic FET-based memory devices, as shown in Figure 2C [72]. AuNPs were encapsulated with alpha-synuclein (αS) protein to form αS–Au NP conjugates, which were absorbed on SiO_2_ to form a tightly packed AuNP monolayer by changing the buffer from pH 6.5 to 4.5 [73]. The thin αS protein layer encapsulating the AuNP with a thickness of ~8.5 nm functions as both a tunneling layer and a blocking layer. Controlling the particle dimension and distribution provides various design alternatives for charge storage characteristics such as current level, hysteresis window, and retention time (Figure 2C). Nishiori et al. demonstrated a light sensor using photosystem I (PSI) linked to a graphene FET via AuNP [74]. The drain current increased upon illumination, showing a photoresponsivity of 4.8 × 10^2^ AW^−1^ and a negative shift of the charge neutrality point by −12 mV. This negative shift under illumination is possibly due to the increase in electron carriers in the graphene induced by a hole trap in the AuNP caused by electron transfer from the AuNPs to PSI. Graphene devices without AuNPs did not exhibit a photoresponse, which confirms that the AuNPs play an important role in charge transfer. Chang et al., employed single-crystal C60 needles (N-C60) and copper phthalocyanine (CuPc) NPs to generate an ambipolar charge-trapping effect to improve the memory window and data storage capacity [75]. A double floating-gate structure was formed in the semiconductor channel (i.e., pentacene) and heterostructured CuPc NPs and N-C60 trapping sites covered by insulating crosslinked poly(4-vinylphenol) (c-PVP). The fabricated double floating-gate device exhibited ambipolar charge-trapping behavior based on the hole trapping in CuPc and the electron trapping in N-C60. The threshold voltage shifts to the negative (positive) direction with hole (electron) trapping. This opposite direction of the threshold voltage shifts resulted in a large memory window.

Some challenges of biomolecule–nanoparticle hybrids such as low field-effect mobility, stability, and reproducibility should be mitigated to exploit their advantages in memory applications. As the electron transfer properties of a biomolecule depend on its orientation, new fabrication methods are necessary to control and align biomolecules with a precise orientation onto devices [76]. Multi-functional devices composed of biosensors and memory devices have considerable potential for various applications. Owing to their unique recognition and catalytic properties, biomolecules can convert biological events via ‘bio-sensing’ to electronic signals, which can be stored in memory devices and further processed. The flexible characteristics of biomolecules and nanoparticles will also pave the way for the application of hybrid nanodevices as wearable electronics.

## 4. Electrochemical Memristor

Among various bioelectronic devices, the biomemristor has been studied extensively in recent years. The memristor, also called resistive memory or resistive random-access memory, is an electrical component in computing systems that performs the role of electrical charge and memory storage. In various scientific fields, memristors have attracted attention because of their wide availability, rapid processing for the memory storage and calculation of logical functions, and low energy requirements [77]. From the perspective of bioelectronics, biomolecules have immense potential as the core components for the development of memristors [78]. In addition, biomolecules demonstrate memristor functions because of their unique properties such as low cost, biocompatibility, lack of requirements for complex chemical synthesis processes, and the ability to perform these functions regardless of a very small size, which provides an opportunity for miniaturization to develop high-density memory and logical applications. Therefore, the development of memristors using biomolecules has recently been reported in the field of bioelectronics. A biomemristor is a type of memristor demonstrated based on the functions of biomolecules.

To date, various biomolecules have been utilized to develop biomemristors, including proteins and nucleic acids. Among various proteins, silk proteins such as the silkworm hemolymph protein and silk fibroin have been extensively studied to demonstrate memristor functions [77,79]. Wen et al. reported a nonvolatile biomemristor device composed of indium tin oxide (ITO), silkworm hemolymph protein, and aluminum layers. Hemolymph protein is widely investigated in entomology and medicine for malaria, as well as dengue fever between insects and humans. The hemolymph protein with an aluminum layer showed non-canonical electrical characteristics that can be applied to the memristor. To fabricate this biomemristor, first, the silkworm hemolymph protein was spin-coated onto the ITO substrate, and the Al layer, which is the upper electrode, was formed on the silkworm hemolymph protein layer as a dot structure with a diameter of 300 μm via thermal evaporation. Cross-sectional scanning electron microscopy, infrared spectroscopy, ultraviolet visible spectroscopy, and cyclic voltammetry (CV) were employed to investigate the properties of the silkworm hemolymph protein and verify the layer formation of the biomemristor. By vertical investigation using the cross-sectional scanning electron microscopy, the layer formation composed of silkworm hemolymph layer and an ITO film layer on a glass substrate was verified. Besides, chemical bonding properties of the silkworm hemolymph were investigated by using infrared spectroscopy, and ultraviolet visible spectroscopy was utilized to obtain the absorption spectra of the silkworm hemolymph protein for calculation of the bandgap width of the silkworm hemolymph protein. Besides, CV was conducted to investigate the electrochemical signals derived from the silkworm hemolymph protein on the substrate. Then, a semiconductor characterization system was used to analyze the current–voltage characteristics of the biomemristor. From the current–voltage analysis, the biomemristor showed excellent memristor properties. From 0 to −5 V, the current value increased suddenly from the OFF state to the ON state, as the writing step. Meanwhile, from 0 to 5 V, the current value decreased suddenly from the ON state to the OFF state, as the erasing step. During the sweeps, this device exhibited write–read–erase–read cycles. In addition, it retained this memristor characteristic for 500 cycles with a high ON/OFF current ratio. In this study, authors successfully developed a nonvolatile rewritable biomemristor device by using the silkworm hemolymph protein with a current switching ratio exceeding 1000. As another example of a biomemristor, a silk-fibroin-based biomemristor device was proposed [79]. In this study, a biomemristor composed of gold, silk fibroin, and platinum layers was developed, and silk fibroin was used as the resistive switching layer between the active and inert electrodes. This biomemristor had excellent biocompatibility when exposed to living cells because of the biocompatible silk fibroin, which was approved by the Food and Drug Administration (FDA) for clinical applications. During the electrical investigation, this silk-fibroin-based biomemristor showed two apparently distinct resistance states. In this system, the gold and platinum layers were utilized as the anode and cathode, respectively. During the voltage sweep from 0 to 5 V, and from 0 to −5 V, this biomemristor exhibited outstanding bipolar resistive switching characteristics with notable changes in the resistance values of 5 × 10^4^ Ω and 0.2 × 10^9^ Ω, respectively. From the results, the proposed biomemristor showed excellent biomemristor characteristics with biocompatibility. Moreover Su et al. developed an egg-albumin-based biomemristor device [80]. Notably, the authors suggested a less expensive method to develop a biomemristor compared with the method used to develop conventional silicon-based memristor devices. In this system, the albumin acquired directly from chicken eggs without further extraction or purification was used as the insulating layer of the device, which was demonstrated to be the most cost-effective method to develop the biomemristor device. In addition, various biomemristors have been reported using biomolecules such as pectin, which are widely contained in plants [81].

**Figure 3:**
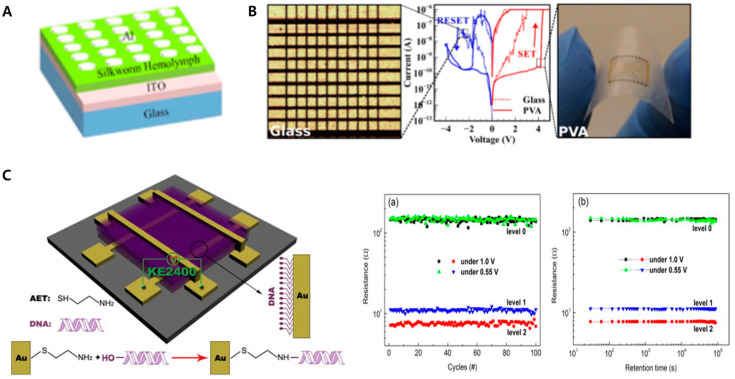
Biomemristor devices based on the proteins: (**A**) nonvolatile biomemristor device composed of the ITO, silkworm hemolymph protein, and an Al layers. used. Reproduced with permission from the authors of reference [77], the figure has followed the terms of use under a Creative Commons Attribution 4.0 International Licenses. (**B**) the resistive switchable biomemristor composed of the gold, silk fibroin, and platinum layers, biomemristor devices based on the DNA. Reproduced with permission from the authors of reference [79], the figure has followed the terms of use under a Creative Commons Attribution 4.0 International Licenses. (**C**) Multilevel biomemristor composed of the various natural DNA layers located between the gold layers with addition of silver ions. Reproduced with permission from [84], published by Elsevier, 2015.

DNA is a suitable biomolecular candidate for developing biomemristors. DNA has some unique intrinsic properties such as a specific binding property with complementary sequences accurately at the nanometer scale, and efficient formation of an insulating layer. These characteristics provide considerable potential for the development of DNA-based biomemristors. Accordingly, studies on the biomemristors prepared using DNA have been conducted recently. Biomemristors composed of gold/DNA/silver layers and silver/DNA/silver layers on silicon dioxide substrates were reported to demonstrate resistive switching functions through an optical waveguide [82]. To achieve this, the DNA layer was sandwiched between two different plasmonic metal layers. Depending on the plasmonic effects, the proposed biomemristor showed apparently distinct current values as ON and OFF states. This study suggested a novel method using an optical waveguide to switch the resistive functions of a DNA-based biomemristor to develop an optical biomemristor. Furthermore, nickel-ion-intercalated DNA nanowires (Ni-DNA nanowires) were used to develop a DNA-based biomemristor [83]. Purified DNA was mixed with buffered nickel ion solutions at a high temperature and then cooled to form the Ni-DNA nanowire. Then, the Ni-DNA nanowire was placed between two different gold terminals on the silicon dioxide substrates via DC electrophoresis. The formation of a Ni-DNA biomemristor was confirmed via atomic force microscopy, and its electrical properties were investigated using an electrometer (Keithley 6517B) under low-humidity conditions. This biomemristor showed resistive switching functions reversibly during the voltage sweeps and retained its characteristics for 700 cycles without loss. Furthermore, a multi-level biomemristor was proposed by using the natural DNA located between the gold layers [84] (Figure 3C). In addition, to demonstrate multi-level memristor functions, various DNA layers were introduced between the gold layers with the addition of silver ions. The prepared biomemristor exhibited three different resistive states: two different ON states under the sweeping voltage amplitudes of 1.0 V and 0.55 V, and the same OFF state for both operation conditions. The authors defined these three different states as Level 1, Level 2, and Level 3, which showed the possibility of development of extremely high-density nonvolatile multi-level biomemristors in the next generation.

In addition, various studies have attempted to develop novel biomemristors. In contrast to electrical or optical-based biomemristors, Katz’s group suggested an electrochemical-based biomemristor device by using laccase enzymes and pyrroloquinoline quinone (PQQ)-dependent glucose dehydrogenase [85]. In addition, to mimic synaptic networks, a nanoscale memristor device was developed for application in bioinspired neuromorphic systems [86]. Thus, novel biomemristors are being actively developed for application in charge storage systems.

## 5. Electrochemical Charge Storage Device

Typically, electrochemical charge storage devices containing biomolecules and nanomaterials have been introduced to modify the redox properties of biomolecules [14,15]. Then, the nanomaterials were used to amplify the electrochemical signal or modulate the redox property. The use of metalloproteins—such as azurin, cytochrome c, ferritin, myoglobin, and hemoglobin—made the redox properties of the biomolecules accessible. For applying metalloproteins to electrochemical charge storage devices, native metalloproteins are required to be immobilized on inorganic substrates such as mercaptoundecanoic acid (11-MUA), mercaptohexanoic acid (6-MHA), mercaptoacetic acid (MAA), and 3,3′-dithiobis (sulfosuccinimidyl propionate) [87,88,89]. Usually, these organic molecules are composed of a head group, chain, and tail group. The head group has a thiol group, which provides the binding site to the Au substrate. The tail group has carboxyl acid, which can bind to the N-terminus of the metalloprotein via a cross-linking agent, such as EDC and NHS. These crosslinking agents enable bonding between metalloproteins and linkers for immobilizing the biomolecule [89,90].

However, these processes require complicated immobilization steps and several reagents. The recombinant azurin was developed by Choi et al. to solve this problem [91]. As a part of the metalloprotein family, azurin contains copper ions, which can store electrons in the redox center. Azurin was modified to introduce cysteine groups via site-directed mutagenesis. The cysteine groups provided the thiol groups on the surface of azurin molecules, which can anchor the Au substrate by covalent bonding directly without an additional linker material. Then, the prepared azurin-modified substrate has redox properties, which were investigated via cyclic voltammetry (CV) to determine the oxidation potential (OP) and reduction potential (RP). When the OP was applied to the recombinant azurin-modified substrate, electrons were stored. In contrast, when the RP was applied to the recombinant azurin-modified substrate, electrons were released from the azurin molecule. Moreover, an open-circuit potential (OCP) technique was employed to evaluate the stored electrons in the azurin molecules. With the OP, RP, and OCP parameters, an electrochemical charge storage device composed of recombinant azurin was achieved. Sequentially, several types of biomemory applications were proposed [92,93,94].

**Figure 4:**
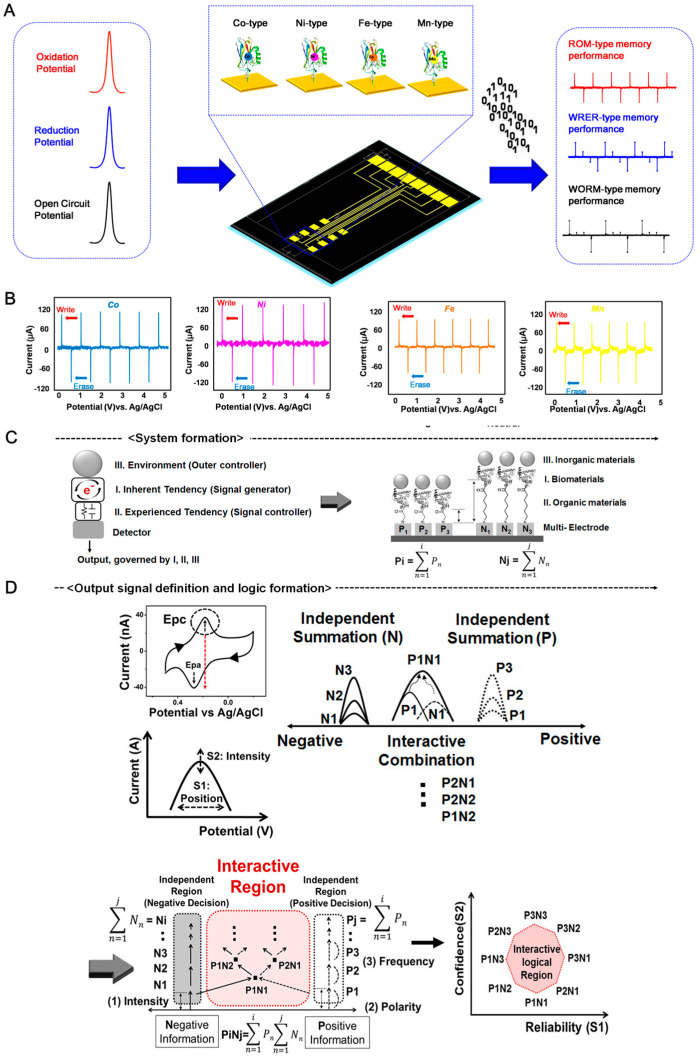
(**A**) Schematic diagram of the basic operating mechanism of the fabricated biomemory chip. Reproduced with permission from [95], published by Elsevier, 2010. (**B**) Verification of the two-state charge storage function by chronoamperometry. Blue line: Co-type azurin, Pink line: Ni-type azurin, Brown line: Fe-type azurin, Yellow line: Mn-type azurin. The figure displays the corresponding charging currents were observed when assigned to oxidation potential and reduction potential repeatedly for a total duration of 5 s. Reproduced with permission from [95], published by Elsevier, 2010. (**C**) Formation of bio-hybrid materials for the realization of proposed concept (left), specific device structure as a hardware-type platform for logic operations (right) [87], the figure has followed the terms of use under a Creative Commons Attribution 4.0 International Licenses. (**D**) Parameter definition and conceptual functions using output results (left), demonstration of defined regions (independent and interactive) using controlled electrochemical signal of metalloprotein (right) [87], the figure has followed the terms of use under a Creative Commons Attribution 4.0 International Licenses.

To tune the functionality of the charge storage device, the metal ion in the metalloprotein can be altered by other ions. Lee et al. suggested the fabrication of a multi-bit biomemory chip consisting of metal-ion-substituted recombinant azurin for performing multi-functional charge storage properties [95]. The copper ion containing azurin was changed to uptake cobalt, nickel, iron, and manganese ions. Each metal-substituted recombinant azurin showed different redox potentials that could be stored at different charge levels in a biomemory chip (Figure 4A). The OPs of Co-type, Ni-type, Fe-type, and Mn-type azurin were determined to be 210.03 ± 15.58 mV, 172.90 ± 16.36 mV, 236.09 ± 21.75 mV, and 132.61 ± 30.54 mV, respectively, and the RPs of Co-type, Ni-type, Fe-type, and Mn-type azurin were determined to be 74.10 ± 20.75 mV, 51.60 ± 7.85 mV, 105.12 ± 15.24 mV, and 24.97 ± 11.26 mV, respectively. The results indicate that the redox properties of azurin can be changed through metal substitution. When OP and RP were applied to Co-type, Ni-type, Fe-type, and Mn-type azurin-modified biomemory chips, the charge storage function was validated via chronoamperometry (CA) (Figure 4B). Thus, the metal-modified azurin layer can be used in charge storage devices.

The functionality of the biomolecule-based charge storage device was improved by introducing various nanomaterials. AuNPs were introduced to the biomolecule-based charge storage device to increase the charged current [96]. Lee et al. developed a biomemory device composed of recombinant azurin and AuNPs. AuNPs of various sizes (5, 10, 20, 40, and 60 nm) were introduced to recombinant azurin-immobilized Au substrates to confirm the electrochemical signal enhancement effect. The enhancement effect was determined via CV and CA. The results showed that 5 nm AuNPs-introduced azurin heterolayer showed the highest charged current. This might be explained by the similar sizes of biomolecules and nanoparticles that can transfer electrons at a 1:1 ratio. Based on the results, the 5 nm AuNP-introduced azurin layer can store three times more electrons than the azurin-modified electrode. This study showed that the nanobio heterolayer could enhance the charge storage function. Moreover, some of reports studied about nanoparticle size-dependent threshold voltage shifts in organic memory transistors [97], renewable juglone nanowires with size-dependent charge storage properties [98], and size-dependent surface charging of nanoparticles [99]. Those studied discussed the size of nanomaterial can affect the charge storage property. Those factors should consider the future nanobio heterolayer-based charge storage device construction.

Furthermore, the assembly of a biomolecule–nanomaterial–organic molecule heterolayer can be used for signal modulation to control the redox potential value and redox current intensity. Chung et al. applied a biomolecule–nanomaterial–organic molecule heterolayer to an analog-type charge storage device for analog decision-making [87] (Figure 4C). Myoglobin was used to perform electrochemical signal generation because of the redox property of the iron ions in the myoglobin. The original redox properties were controlled using various chemical linkers (2-MAA, 3-MPA, 6-MHA, 8-MOA, 11-MUA, and 16-MHA). The gap size between myoglobin and the Au substrate can be controlled using various linkers. Each linker had the different carbon number in the backbone structure. Presumably, those different molecular lengths hampered the electron transfer between protein/nanoparticle and substrate at the nanoscale. In addition, nanomaterials such as AuNPs, QDs, and metal ions were used for signal modulation. The nanomaterial/biomolecule/chemical linker heterolayer is composed of an electrochemical logic unit. The S1 area was defined as a potential-based peak position that resulted from polarity and the S2 area was defined as the current-based peak intensity caused by the cathodic peak value of myoglobin. These electrochemical values were used as the outputs of the logical decision. Thus, the combination of biomaterials, nanomaterials, and organic molecules can be applied to electrochemical logical devices based on charge storage modulation. These parameters varied with the environmental factors, e.g., organic spacers immobilized on metal surfaces, inorganic materials bound on metalloproteins, and combinations of input information. Then, multi-electrode patterns (input frequency) were used as a solid platform for the selective immobilization of metalloproteins based on reductive cleavage. A functional charge storage device that showed an environment-dependent interactive decision-making process by signal convergence with overlapping and combining negative and positive recognition was fabricated (Figure 4D). To construct an interactive logic region, a single output signal needs to provide the specific quantity and polarity of charge inputs from various multi-electrodes. Another idea was to obtain a single electrochemical output by merging the output signals from two different inputs (positive and negative). To achieve this, positive and negative input combinations were set in the multi-electrode using an organic spacer set and nanoparticles. The final output signal could be regulated by the ratio of positive to negative information. Thus, the biomolecule–nanomaterial–organic molecule heterolayer can be used to construct a functional charge storage system.

## 6. Conclusions and Future Perspectives

So far, nanobiotechnology has been applied in various fields including pharmaceuticals, biomedical engineering, biosensors, in vitro diagnostics, drug delivery, and point-of-healthcare systems. Furthermore, nanobiotechnology has immense potential for application to new types of charge storage in the era of the Fourth Industrial Revolution.

Charge storage devices are widely applied in numerous industrial fields, such as smartphones, electronic vehicles, medical devices, and electrical devices. The miniaturization ability and high capacity of charge storage devices have been studied by companies and research groups worldwide. However, the use of biomolecule–nanomaterial hybrids for application to charge storage devices has not been studied yet. The unique features of biomolecules have various functionalities. For example, metalloproteins have redox properties, the enzyme shows enzymatic kinetics to the target substrate, and the antibody has a specific affinity to the target antigen. Furthermore, nucleic acids have various properties. Single-strand nucleic acids hybridize with complementary sequences, and the DNAzyme and ribozyme show enzymatic properties that cannot be achieved using conventional enzymes. The nucleic acid aptamer can bind the target protein, DNA, or other supports. These biomolecules can be combined with hundreds of nanomaterials.

Compared with biomolecules, there are infinite possibilities for the synthesis of unknown nanomaterials. Several scientists and engineers have attempted to develop novel nanoparticles having various advantages such as low toxicity, high conductivity, low manufacturing cost, and biocompatibility. In this review, we have examined AuNPs, Ag nanoparticles, Pt nanoparticles, QDs, graphene, topological insulators, and carbon nanotubes in combination with biomolecules. At this stage, we cannot evaluate which nanoparticle–biomolecule combination shows the best charge storage performance. In the future, we will determine the combination showing the best storage capacity, cost, toxicity, and compatibility. Furthermore, the commercialization issues should be solved to conduct the nanobio heterolayer-based charge storage device. The current lithium-ion battery (LIB) system composed of organic molecules is widely used nowadays, the combination of organic molecule was introduced to increase the stability, charge storage, and affordability. However, compared to LIB, the nanobio heterolayer-based charge storage device should solve several problems, such as (1) mass production of nanomaterial, (2) mass production of biomaterial, (3) stability problems, and (4) performance tuning.

As shown in this review, the biomolecule–nanomaterial heterolayer can be integrated into living organisms and shows unprecedented characteristics that cannot be achieved using conventional charge storage devices. For example, glucose oxidase and carbon nanotube heterostructure can be operated the biofuel cell function in living snail [100]. Also, the enzyme-based biofuel cell system operated in the living lobster [101]. That can be applied to living battery and heart pacemaker. However, ethical problems and efficiency issues should be considered for future work. Thus, we believe that this new concept of a charge storage device composed of a biomolecule–nanomaterial heterolayer has immense potential in various applications.

The charge storage devices composed of a biomolecule–nanomaterial heterolayer discussed in this review such as biomemristors, FETs, and biomaterials can be simply applied in the near future. The next objective is the realization and commercialization of these charge storage devices. We cannot predict the future size, capacity, weight, and purpose of these charge storage systems, but we can imagine that these charge storage devices will be integrated into the human body or living organisms in the near future.
